# Lead remediation is promoted by phosphate-solubilizing fungi and apatite *via* the enhanced production of organic acid

**DOI:** 10.3389/fbioe.2023.1180431

**Published:** 2023-03-30

**Authors:** Da Tian, Xiaoru Zhang, Liyan Wang, Mingxue Han, Chaochun Zhang, Xinxin Ye

**Affiliations:** ^1^ Anhui Province Key Laboratory of Farmland Conservation and Pollution Prevention, College of Resources and Environment, Anhui Agricultural University, Hefei, China; ^2^ Anhui Province Engineering and Technology Research Center of Intelligent Manufacture and Efficient Utilization of Green Phosphorus Fertilizer, Anhui Agricultural University, Hefei, China; ^3^ Key Laboratory of JiangHuai Arable Land Resources Protection and Eco-Restoration, Ministry of Natural Resources P. R. C, Anhui Agricultural University, Hefei, China

**Keywords:** lead remediation, apatite, phosphate solubilizing fungi, organic acid, phosphorus release

## Abstract

Lead (Pb) is one of the most common heavy metal pollutants in the environment, which can indirectly or directly threaten human health. Lead immobilization by apatite can reduce the effectiveness of Pb cations *via* the formation of pyromorphite (Pyro). However, the formation of Pyro is always depending on the release of phosphorus (P) from apatite. Phosphate-solubilizing fungi (PSF) can secrete large amounts of organic acid to promote the release of P from apatite. Although the combination of PSF and apatite has shown a huge potential in Pb remediation, this pathway needs to be more attention, especially for organic acid secretion by PSF. This research mainly reviews the possible pathway to strengthen Pb immobilization by PSF and apatite. Meanwhile, the limitation of this approach is also reviewed, with the aim of a better stabilizing effect of Pb in the environment and promoting the development of these remediation technologies.

## 1 Introduction

Lead (Pb) is one of the most important heavy metal pollutions in the environment, which has strong biological toxicity, wide distribution, and strong accumulation capacity (Arduini et al., 2010). The completely remove of Pb cations from soil is relatively long and complex due to the hidden and lagging performance (Shen et al., 2015). In-suit immobilization of Pb is an efficient pathway to reduce the toxicity of Pb in soil (Chen et al., 2006). Phosphate can effectively transfer Pb cations to highly insoluble Pb minerals *via* the phosphorus (P) release (Li et al., 2016b; Tian et al., 2018). However, the process of P release is unsustainable and easily chelates with metal cations in soil, e.g., Ca^2+^, Fe^3+^, etc (Tian et al., 2021a). The combination of phosphate solubilizing fungi (PSF) and phosphate is an effective and sustainable pathway in Pb in suit immobilization (Shao et al., 2021). As a new approach in Pb remediation, this technology needs to be more attention nowadays.

## 2 Mechanism of Pb remediation by phosphate solubilizing fungi and phosphate

**FIGURE 1:**
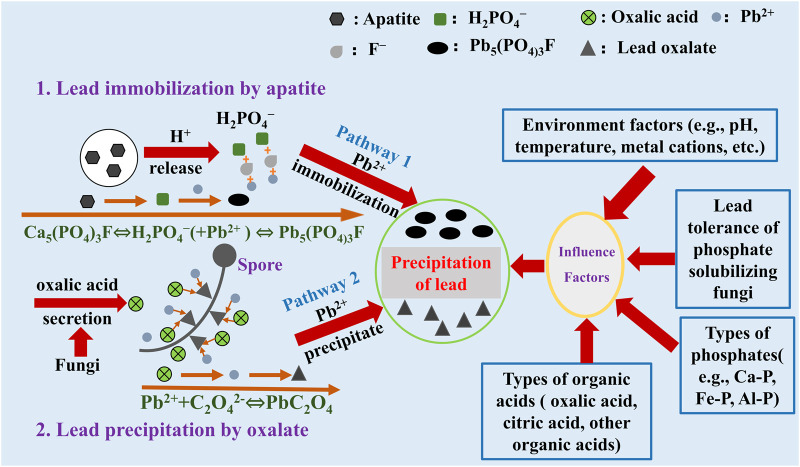
Lead remeditaion by phosphate solubilizing fungi and phosphate *via* the secretion of organic acid.

In current, phosphate is generally recognized as an excellent material in Pb remediation. The P released from phosphate can react with Pb to form highly insoluble pyromorphite (Pyro) Figure 1. Pyro is highly stable and has a low K*sp* value (<10^–85^), which can significantly reduce Pb toxicity and mobility in soil (Arnich et al., 2003; Chaturvedi et al., 2007; Cheyns et al., 2012). In engineering, field and indoor leaching simulation tests, phosphates can convert Pb from high-activity forms to insoluble forms (John et al., 2001; Chen et al., 20231). The application of phosphate can reduce 12%–92% available Pb content in soil, and the toxicity characteristic leaching procedure Pb (TCLP-Pb) concentration can decrease from 82 mg/L to less than 5 mg/L (Melamed et al., 2003; Cao et al., 2009; Cui et al., 2010). The reaction formula between P and Pb is as follows (Park and Bolan, 2013):

$$10{Pb}^{2+}+6H_{2}{PO}_{4}^{-}+2X^{-}={Pb}_{10}{\left({PO}_{4}\right)}_{6}X_{2}+12H^{+}\left(X=OH,Cl,F\right)\;\left(Formation\;of\;stable\;Pb\;minerals\right)\text{(1)}$$

An acidic environment can significantly enhance phosphate dissolution and improve the release of P (Whitelaw, 1999; Singh and Reddy, 2011). However, the addition of chemical acid (e.g., sulfuric acid) is unsustainable and harmful to soil health. Oxalic acid is more efficient than sulfuric acid in phosphate dissolution (Mendes et al., 2020). Therefore, the utilization of oxalic acid and phosphate is a better choice in Pb remediation. Phosphate solubilizing fungi (PSF) can secrete large amounts of oxalic acid and promote the release of P from insoluble phosphate (Li et al., 2016a). Compare with bacteria, PSF not only maintains the ability of oxalic acid secretion but also can extend in soil *via* the mycelium. For example, the PSF of *Aspergillus niger* can promote the dissolution of FAp and carbonate in soil *via* the hypha extension and oxalic acid secretion (Tian et al., 2021b). In addition, the PSF of *A. niger*, *Penicillium oxalicum,* and *Penicillium aurantiogriseum*, etc., also has a strong ability to secrete oxalic acid (Tian et al., 2021a; Hu et al., 2022; Wang et al., 2022). Therefore, the combination of PSF and phosphate is a considerable pathway in Pb remediation.

The application of PSF and phosphate have been successfully applied in Pb remediation. *A. niger* and *P. oxalicum* combined with FAp can significantly remove more than 90% Pb cations in an aqueous solution *via* the formation of lead oxalate and Pyro (Li et al., 2016b; Tian et al., 2018). Meanwhile, Pb remediation in soil by this combination not only promotes the formation of lead oxalate but also increased the soil available P content (Tian et al., 2022b; Meng et al., 2022). In addition, the released P can be also isolated by PSF and not easily absorbed by plants, promoting the Pb remediation process (Menezes-Blackburb et al., 2016). However, the secretion of oxalic acid by PSF is usually influenced by different factors, such as pH, nutrients, phosphate types, and Pb concentration (Tian et al., 2019; Feng et al., 2022). Therefore, the appropriate technology and conditions are needed in Pb remediation by PSF and phosphate.

## 3 Effect factors in Pb remediation by PSF and phosphate

### 3.1 Pb tolerance of phosphate solubilizing fungi

Pb remediation by PSF is usually affected by different Pb toxicity. The excessive concentration of Pb cations can limit the growth of fungi and reduce their bioremediation efficiency (Ye et al., 2018). However, the tolerance of Pb toxicity in PSF is different. *A. niger* has a higher Pb tolerance than *P. oxalicum* (Tian et al., 2018; Tian et al., 2019). *A. niger* can survive under 1,500 mg/L Pb concentration and maintain the ability of oxalic acid secretion (Tian et al., 2019). However, the PSF of *P. oxalicum* only resists 1,000 mg/L Pb concentration, and the secretion of oxalic acid is almost lost under 1,500 mg/L Pb concentration (Tian et al., 2019). Therefore, *A. niger* has a high Pb tolerance and is efficient in Pb remediation.

### 3.2 Suitable phosphate types in Pb remediation

The type of phosphate affects the efficiency of Pb remediation mainly due to the P release capacity (Tian et al., 2021a). Hence selecting an appropriate phosphate is important in Pb remediation. The use of phosphates in Pb remediation usually contains water-soluble phosphates (WSP) and insoluble phosphates (IPs), including potassium dihydrogen phosphate, sodium dihydrogen phosphate and hydroxyapatite, fluorapatite bioapatite, etc. WSP has a high solubility of P and is efficient in Pb remediation. However, the use of WSP is easy to cause eutrophication of water and the excessive P can be fixed by metal cations in soil. Compared with WSP, IPs are more stable and need to mix with PSF in Pb remediation (Li et al., 2016b). PSF combined with IP can promote the continuous release of P *via* the secretion of organic acid and is suitable for long-term Pb remediation. However, the different IPs can affect the secretion of organic acid by PSF. For example, calcium phosphate (Ca-P) can stimulate *A. niger* to secrete more oxalic acid (Tian et al., 2021a). In addition, the dissolution of Ca-P is more efficient than Fe-P by *A. niger*. Therefore, Ca-P is the best choice in Pb remediation by PSF.

### 3.3 Effects of nutrients on PSF in Pb remediation

The different nutrients can significantly influence the secretion of organic acid by PSF and hence affect Pb remediation by phosphate. In the case of oxalate, the secretion of oxalate by PSF is affected by different environmental factors, such as carbon (C) source, nitrogen (N) source, environmental pH, etc (Palmieri et al., 2019). Nitrogen is a key factor affecting the metabolism of *A. niger* and the dissolution of phosphate rock (Paulo et al., 1988; Tian et al., 2018). Compare with ammonium and urea, nitrate can significantly increase the secretion of oxalate by *A. niger* and reduce the Pb concentration in Pb remediation with Ca-P (Feng et al., 2022). For nitrogen, nitrate is the suitable resource in Pb remediation by PSF and phosphate.

## 4 Ways to improve lead remediation

### 4.1 Application of fertilizers in Pb remediation

PSF and phosphate complex have been used to produce phosphate-based biofertilizers, which not only increase the P content in the soil but also function in Pb remediation (da Silva et al., 2017). The application of PSM biofertilizer can significantly increase crop yield and soil available P content, reducing the 50% phosphate fertilizer input (Fitriatin et al., 2017). Phosphate rock combined with PSF (*P. oxalicum*) can replace chemical fertilizers, and increase crop yield. In addition, the application of PSM biofertilizer and phosphate can also reduce the Pb concentrations in soil. For example, the combination of phosphogypsum (PG) and biofertilizer (containing *A. niger*) can reduce soil Pb concentration from 365 mg/kg to 302 mg/kg (Meng et al., 2022). PG not only provide a sufficient P source for the growth of *A. niger* in highly contaminated soils but also strengthens the formation of insoluble Pb minerals. Therefore, adding phosphate and PSF as fertilizer is an effective attempt at long-term Pb remediation.

### 4.2 Application the suitable nutrients

Nitrogen sources can significantly affect the secretion of organic acids of *A. niger*, which could affect phosphate dissolution and Pb remediation (Gadd et al., 2014). The decomposition of inputted urea can produce carbon dioxide and form carbonates, which inhibits the growth of *A. niger* and the secretion of organic acids (Cinthya et al., 2006; Su et al., 2021). Ammonium and nitrates are more efficient in Pb remediation by *A. niger* and phosphate (Feng et al., 2022). In addition, calcium can stimulate *A. niger* to secrete more organic acids, hence the calcium-based nitrogen fertilizer is more suitable for Pb remediation by PSF and phosphate (Tian et al., 2021a). In addition, other microorganisms such as *Rhodotorula mucilaginosa* (Rho) can secrete large amounts of extracellular polymers (EPS) to form EPS-Pb in Pb toxicity resistance (Li et al., 2019). The addition of phosphate can significantly promote the secretion of EPS by Rho (Tian et al., 2022a). The Pb remove ratio in Rho and phosphate reached 99.9% (Tian et al., 2022a). In addition, the polysaccharides and other nutrients contained in EPS can support the growth of PSF. Therefore, EPS can be applied as a synergist in Pb remediation by PSF and phosphate.

## 5 Discussion

In summary, the combination of PSF and phosphate in Pb remediation is an effective way in current research. On the one hand, PSF can secrete oxalic acid to promote the release of P from phosphate, and the released P can react with Pb cations to form highly insoluble pyromorphite. On the other hand, the secreted oxalic acid by PSF can also react with Pb to form insoluble lead oxalate Figure 1. However, this pathway is also limited due to the long-time dissolution of phosphate and the formation of insoluble Pb minerals. Increasing the secretion of oxalic acid by PSF is the key factor in Pb remediation by the combination of phosphate. Hence, the ability of oxalic acid secretion by PSF should be considered in a different environment. In the future, the enhancement of the micro-interface process in Pb remediation by PSF and phosphate should be explored, especially in strengthening the participation of oxalic acid. Improving the production of oxalic acid *via* the different pathways can promote Pb remediation faster and completely to reduce Pb toxicity. In addition, to obtain the best Pb remediation purpose in the environment, choosing the suitable PSF and phosphate are needed in practical application.

## Data Availability

The original contributions presented in the study are included in the article/supplementary material, further inquiries can be directed to the corresponding authors.
